# Prenatal Ultrasound Diagnosis of Binder Phenotype: Case Series of Seven Patients and Literature Review

**DOI:** 10.3390/reports8030188

**Published:** 2025-09-22

**Authors:** Silvia Andrietti, Alessia Maccarrone, Giuseppe Gullo, Valentina Billone, Lina De Paola, Chiara Gaggero, Diliana Beleva, Chiara Calcagno, Pierangela De Biasio

**Affiliations:** 1 Prenatal Diagnosis and Perinatal Medicine Unit, IRCCS Ospedale Policlinico San Martino, 16132 Genova, Italy; silvia.andrietti@gmail.com (S.A.); chiara.gaggero@hsanmartino.it (C.G.); diliana.beleva@hsanmartino.it (D.B.); chiara.calcagno2@hsanmartino.it (C.C.); pierangela.debiasio@hsanmartino.it (P.D.B.); 2 Department of Neurology, Rehabilitation, Ophtalmology, Genetics, Maternal and Infant Health (DiNOGMI), 16132 Genoa, Italy; alessia.maccarrone97@gmail.com; 3 Department of Obstetrics and Gynaecology, Villa Sofia Cervello Hospital, University of Palermo, 90146 Palermo, Italy; valentina.billone@gmail.com; 4 Department of Anatomical, Histological, Forensic and Orthopedic Sciences, Sapienza University of Rome, 00161 Rome, Italy; lina.depaola@uniroma1.it

**Keywords:** binder phenotype, binder syndrome, maxillonasal hypoplasia, midfacial hypoplasia, first trimester screening, case series, facial dysmorphism, abnormal fetuses

## Abstract

**Background and Clinical Significance**: Binder syndrome or maxillonasal dysplasia is a rare developmental disorder affecting the anterior maxilla and nasal complex, characterized by midfacial hypoplasia, a flattened nasal bridge, and increased nasofrontal angle. **Case Presentation:** We present a case series of seven fetuses diagnosed with Binder phenotype through targeted ultrasound examination at our prenatal diagnosis center during the SARS-CoV-2 pandemic, between September 2021 and July 2023, including the first case described in the literature before 14 weeks. The median gestational age at diagnosis was 21 weeks. Ultrasound features included flattened fetal facial profile, increased nasofrontal angle (>143°), verticalized nasal bones and widened maxillary alveolar arch. Five cases presented as isolated anomalies, while two showed associated findings including growth restriction and polyhydramnios. Invasive prenatal diagnosis was offered in all cases, with three patients consenting to amniocentesis, all revealing normal karyotype and chromosomal microarray. Pregnancy outcomes varied: three patients opted for termination of pregnancy, one case resulted in intrauterine fetal demise, one delivered prematurely with confirmed postnatal phenotype, and two continued pregnancy with normal delivery. **Conclusions**: This relatively high case frequency within a short timeframe suggests that Binder syndrome, while rare, may not be as uncommon as previously reported. Accurate ultrasound diagnosis combined with comprehensive genetic counseling enables appropriate pregnancy management and optimal perinatal outcomes.

## 1. Introduction and Clinical Significance

Maxillo-nasal dysplasia, or Binder’s Syndrome, is a rare developmental disorder of the central portion of the splanchnocranium, characterized by hypoplasia of the maxilla and nasal complex.

The actual incidence is unknown, but it is estimated at 1/18,000 births [1,2]. The true prevalence may be underestimated, as milder phenotypic presentations can remain undiagnosed or be misclassified.

In 1962, Binder et al. [3] defined the phenotypic characteristics that outline a particular facial dysmorphism: a short nose with a flat nasal bridge, the absence or hypoplasia of the anterior nasal spine and columella, an acute naso-labial angle (76–88° vs. normal value 103–117°) with pronounced convexity of the upper lip, prognathism with a tendency toward class III malocclusion due to maxillary hypoplasia, and mild hypertelorism.

Binder syndrome is a composite phenotype rather than a single entity [4], and the cause of Binder phenotype is heterogeneous. The etiology has been categorized into three groups: (1) isolated, (2) associated with vitamin K deficiency/maternal conditions like systemic lupus erythematosus, (3) syndromic [5].

Several publications indicate that ultrasound can detect the characteristic facial features of Binder phenotype before birth [6,7,8]. The sonographic antenatal diagnosis is based on the observation of three key facial features during the second or third trimester: verticalized nasal bones, a flattened nose, and retrusion of the maxilla [6]. Once the prenatal diagnosis of BP is made, a careful fetal anatomical evaluation must be performed along with prenatal invasive testing, since the prognosis may vary according to the underlying causes [7,9,10,11].

Binder condition in the first trimester has not yet been described [2].

The aim of this study is to present a case series of fetuses with BP diagnosed by ultrasound in a short timeframe during the SARS-CoV-2 pandemic, including the first case described in the literature before 14 weeks. We also conducted un updated review of the literature.

## 2. Case Presentation

We present retrospectively a case series of seven fetuses diagnosed with Binder phenotype through targeted ultrasound examination at our fetal medicine unit between September 2021 and July 2023, as shown in Table 1. A database search was performed in April 2025 from computerized medical records. The keywords “abnormal profile,” “flat profile,” and “suspected Binder phenotype” were used.

### 2.1. Case 1

A 25-year-old gravida 1 para 0 woman was referred to our fetal medicine unit at 21 weeks and 1 day of gestation. Her anomaly scan revealed abnormal facial features.

The pregnancy was conceived spontaneously.

The patient’s medical history includes chronic hypertension and a prior hospitalization for cholelithiasis. Moreover, she was experiencing severe hyperemesis gravidarum. At the time of referral, she was receiving treatment with ursodeoxycholic acid, labetalol, and low-dose aspirin 150 mg. Details required for the study, including maternal age, gestational age, maternal BMI before pregnancy, diabetes, hypertension, family history of congenital anomalies, consanguinity, smoking, alcohol consumption, and folic acid assumption, were collected from medical records.

**Figure 1:**
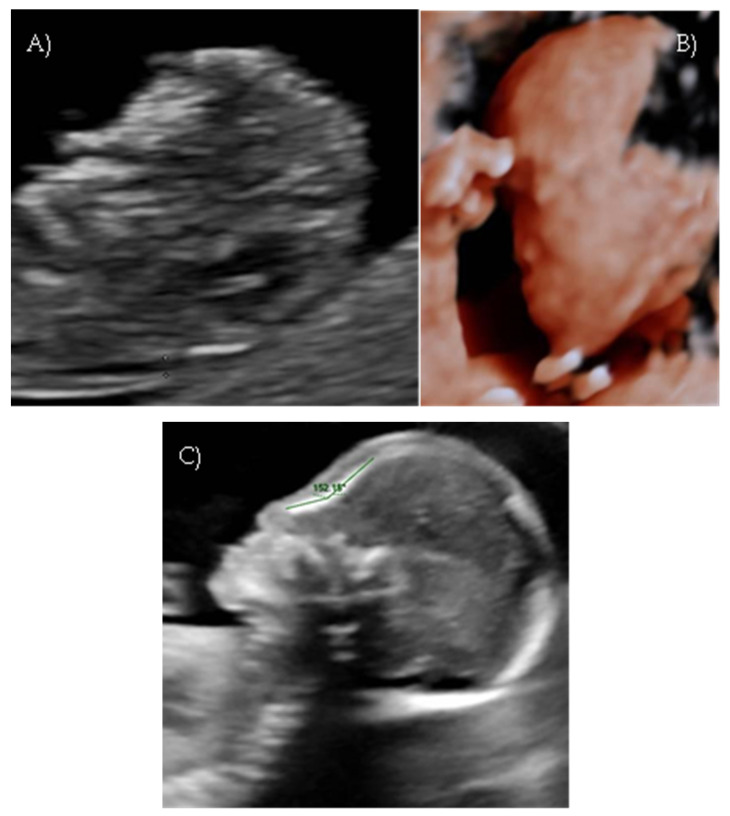
*Case 1*. (**A**) First-trimester 2D ultrasound Fetal profile. (**B**) Flat fetal profile, 21 weeks and 1 day, 3D reconstruction. (**C**) Nasal-frontal angle: 152.18°.

Her first-trimester combined screening for aneuploidies was low risk for trisomy 21, 18, and 13, and preterm preeclampsia. The nuchal translucency was within normal ranges (Figure 1A).

A detailed ultrasound scan was performed at 21 weeks and 1 day of gestation at our fetal medicine unit. Nasofrontal angle, nasal bone, and maxillary width were measured and compared to published references to unaffected fetuses at similar gestational ages [12,13]. Facial dysmorphism was further investigated by 3D scan (Figure 1B) through multiplanar mode and surface rendering, which allowed a more realistic representation of the fetal face, thus allowing characterization of the defect.

A flattened fetal facial profile with a nasal-frontal angle of 152.18° (cut-off: <143°, [14]), consistent with Binder-type maxillonasal dysplasia, was confirmed. To identify any other structural abnormalities, detailed examinations of the fetal anatomy and a fetal echocardiogram were conducted. No additional structural anomalies were identified (Figure 1C).

Given the ultrasound findings and a family history of craniofacial anomaly, invasive prenatal testing, including amniocentesis with karyotype and array CGH analysis was recommended. Genetic counseling was also included to explain the results and their implications for the index pregnancy and future pregnancies.

However, the couple declined amniocentesis and opted to continue the pregnancy.

Serial ultrasound scans revealed no other evolving abnormalities, fetal growth remained appropriate, with normal biometric parameters, and the pregnancy continued to term.

The patient was admitted at 40 weeks and 3 days of gestation for labor induction due to gestational diabetes (managed with dietary modification) and maternal hypertension. Labor induction was initiated with a cervical ripening balloon (CRB) and then continued with dinoprostone the following day. Labor progressed to spontaneous vaginal delivery in the evening, with episiotomy performed due to non-reassuring cardiotocographic patterns. A male infant was delivered with a birth weight of 3210 g and Apgar scores of 9 at 1 min and 10 at 5 min. The facial dysmorphism observed during prenatal development was confirmed after birth.

### 2.2. Case 2

We present a case of a 30-year-old multipara who underwent a first-trimester combined screening test for aneuploidies and preterm preeclampsia at our fetal medicine unit at 13 weeks. The pregnancy was conceived spontaneously.

Her medical history included first-trimester hyperemesis gravidarum and no folic acid supplementation during early pregnancy. The first-trimester combined screening was low risk for trisomy 21, 18, and 13 and preterm preeclampsia.

**Figure 2:**
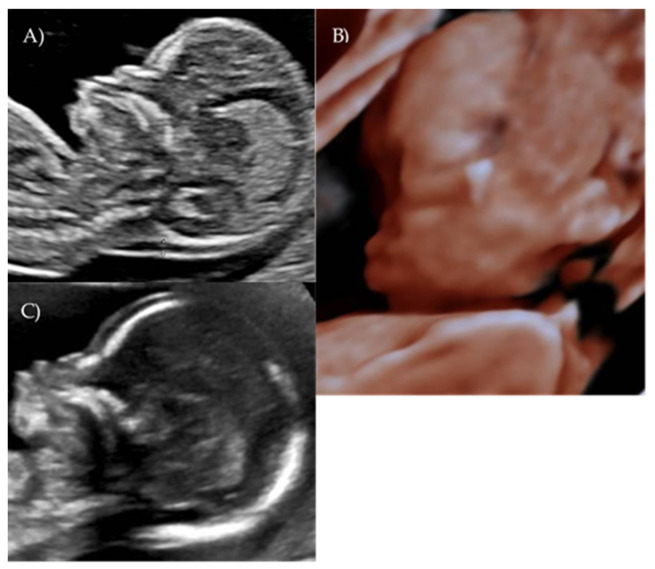
*Case 2.* (**A**) First-trimester 2D ultrasound fetal profile. (**B**) Flat fetal profile, 3D reconstruction, at 17 + 4 w. (**C**) Flat fetal profile, 17 + 4 w.

During the first-trimester scan a flat fetal profile was noted and suspected of indicating a fetal abnormality, and early re-evaluation of the fetal craniofacial anatomy was recommended (Figure 2A).

At 17 weeks and 4 days of gestation, follow-up ultrasound confirmed a flattened fetal facial profile consistent with Binder-type maxillonasal dysplasia (Figure 2B,C). Other fetal anatomy findings appeared normal.

Due to ultrasound features, genetic analysis was recommended. Those included amniocentesis with standard karyotyping, quantitative fluorescence PCR (QF-PCR), chromosomal microarray analysis (array CGH), and DNA storage. Genetic counseling was also advised.

At 18 weeks and 3 days the patient underwent amniocentesis: the fetal standard and molecular karyotype were normal.

Next-Generation Sequencing (NGS) and exome sequencing were discussed with the patient. However they were not yet available for prenatal use in the specific local laboratory during this particular timeframe The costs of NGS and exome sequencing if performed in a different region were not covered by the healthcare system and the patient declined further investigations due to the high cost.

A multidisciplinary approach involved specialists like obstetricians, perinatologists, fetal medicine specialists, neonatologists, orthodontists, and oromaxillary surgeons. This collaborative effort ensured comprehensive assessment, accurate diagnosis, and appropriate management, addressing both the fetal and postnatal needs.

Following diagnosis, the patient opted for pregnancy termination 19 weeks and 3 days.

Fetal autopsy confirmed hypoplasia of nasal bones and cartilages with significant nasal flattening, a prominent philtrum, short neck, and prominent frontal bosses. No additional somatic or skeletal malformations were identified. The fetus was male. The findings were consistent with a maxillofacial malformation pattern of likely syndromic significance.

### 2.3. Case 3

A 29-year-old gravida 3 para 2 woman was referred at our fetal medicine unit at 21 weeks and 2 days of gestation for second opinion on abnormal facial profile.

Her medical history included total thyroidectomy treated with thyroxin and hyperemesis gravidarum in the first trimester.

The first-trimester combined screening for aneuploidies was low risk for trisomy 21, 18, and 13.

An anomaly scan revealed monolateral pyelectasis non-visualization of the stomach bubble, and suboptimal evaluation of the fetal heart’s left long axis.

**Figure 3:**
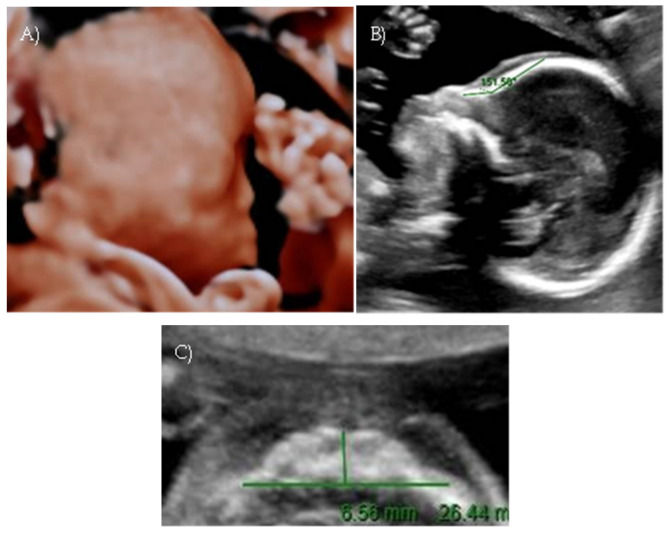
*Case 3.* (**A**) Flat fetal profile, 3D reconstruction. (**B**) Nasal-frontal angle: 151.50°. (**C**) Flattening of the curvature of the maxillary alveolar processes. Width: 26.44 mm.

A targeted second-level ultrasound at our center revealed a flat fetal facial profile: the nasal-frontal angle measured 151.50°, and flattening of the curvature of the maxillary alveolar processes was noted (Width: 26.44 mm, Cut-off at 21 weeks: 23.1 mm [6]) suggestive of Binder-type maxillonasal dysplasia, moderate monolateral calyceal-pelvic dilatation of 8 mm in the left kidney, two echogenic intracardiac foci, and long bone biometry at the lower limits of normal (Figure 3 A–C).

Having detected these abnormalities, genetic investigations were recommended. Following consent, amniocentesis was performed on the same day. Samples were analyzed for standard karyotype, QF-PCR, and chromosomal microarray (CGH-array), with DNA preserved.

Next-Generation Sequencing (NGS) and exome sequencing were discussed with the patient. However the patient declined further investigations due to the high cost.

Fetal echocardiography and genetic counseling were also recommended.

Results of the invasive testing revealed a normal male karyotype and a normal CGH-array. The couple decided to deliver the baby at a different hospital. A female neonate weighting 3.2 kg was born with a vaginal delivery. Respiratory distress and feeding difficulties were reported on neonate’s clinical notes.

### 2.4. Case 4

A 38-year-old gravida 2 para 1 woman was referred at 26 weeks of gestation following concerns during a routine fetal growth assessment. Her medical history included gestational diabetes managed with dietary therapy and the presence of uterine fibroids.

The first-trimester combined screening for aneuploidies was low risk for trisomy 21, 13, and 18. A mid-trimester anomaly scan was reported as normal.

At 26 weeks, fetal biometry showed an estimated weight in the 28th percentile, but all long bone measurements were below the 5th percentile, raising suspicion for skeletal dysplasia versus early-onset intrauterine growth restriction (IUGR).

Genetic testing through invasive diagnosis was recommended but declined. A fetal echocardiogram was normal, and genetic counseling advised follow-up with serial level II ultrasounds.

Close ultrasound monitoring was performed, with regular checks of fetal biometry and amniotic fluid volume, every 2 to 4 weeks.

Subsequent evaluations confirmed persistent fetal growth restriction, with overall biometry at the 10th percentile and long bones remaining below the 5th percentile.

**Figure 4:**
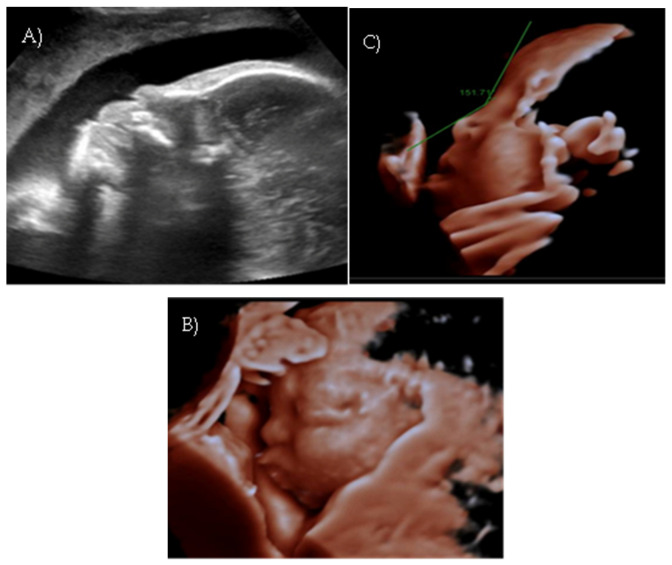
*Case 4.* (**A**) Flat fetal profile. (**B**) 3D reconstruction of the facial profile. (**C**) Nasal-frontal angle: 151.71°.

Polyhydramnios developed; a flat fetal facial profile with mild verticalization of the nasal bone (Nasal-frontal angle: 151.71°, cut-off: <143°) and slight prognathism were noted (Figure 4A–C).

Serial ultrasound scans were performed every 2 to 4 weeks to monitor fetal growth and amniotic fluid volume. At 32 weeks, corticosteroids were administered for pulmonary maturation.

At 35 weeks of gestation, the patient underwent emergency cesarean delivery due to preterm premature rupture of membranes (PPROM) and breech presentation.

A live female infant was delivered. Physical examination revealed a distinctive facies characterized by a flat nasal bridge, bilateral preauricular pits, and a tendency to maintain the tongue in a protruded position. Postnatal karyotype was performed and found to be normal.

### 2.5. Case 5

A 49-year-old gravida 2 para 0 woman was referred at 21 weeks and 4 days of gestation for a second opinion regarding abnormal fetal facial profile. The pregnancy resulted from an intracytoplasmic sperm injection (ICSI) with donor oocytes (donor aged 29 years). Her medical history included mixed connective tissue disease under treatment with hydroxychloroquine and low-dose aspirin.

Her first-trimester combined screening was low risk for trisomy 21, 18, and 13. She was referred after a second-trimester screening ultrasound raised concerns about a flat fetal facial profile.

**Figure 5:**
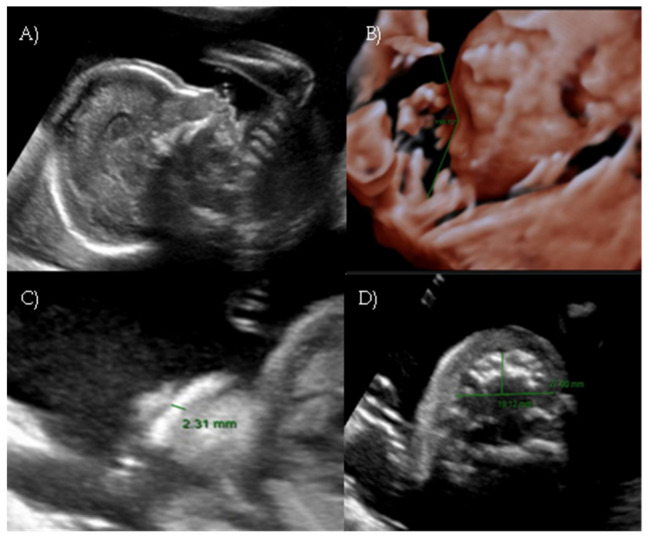
*Case 5*. (**A**) Flat fetal profile with verticalization of the nasal bone. (**B**) Nasal-frontal angle: 150.72°. (**C**) Absent nasolabial philtrum. (**D**) Flattening of the curvature of the maxillary alveolar processes. Width: 27 mm.

Targeted ultrasound at our center confirmed a flat profile suggestive of Binder-type maxillonasal dysplasia with a nasal-frontal angle of 150,72° (cut-off: <143°), flattening of the curvature of the maxillary alveolar processes (Width: 27 mm, cut-off at 21 weeks: 23.1 mm), short columella, and absent nasolabial philtrum (Figure 5A–D). The remaining fetal anatomy appeared normal.

Genetic investigations were recommended, including amniocentesis with standard karyotype, QF-PCR, chromosomal microarray (CGH-array), and DNA storage. Next-Generation Sequencing (NGS) and exome sequencing were discussed with and declined by the patient due to the high cost.

A fetal echocardiogram and genetic counseling were also advised.

The patient opted to proceed with invasive prenatal diagnosis and subsequently chose termination of pregnancy.

Proper patient information has proven to be crucial. It must never be forgotten that this is an integral part of the care process [15,16], in addition to helping prevent medico-legal disputes [17,18,19].

Results of the invasive testing revealed a normal female karyotype and a normal CGH-array.

Fetal autopsy confirmed a female fetus with severe hypoplasia of the maxillary bones, markedly flattened nose, and hypertelorism. No other major macroscopic malformations were identified.

The craniofacial abnormality was consistent with maxillonasal dysplasia (Binder type).

### 2.6. Case 6

A 36-year-old primigravida with no other comorbidities was referred at 22 weeks of gestation for a second opinion regarding the fetal spine, the posterior cranial fossa, and the upper lips following a routine second-trimester ultrasound performed elsewhere.

First-trimester combined screening was low risk for trisomy 21, 13, and 18.

**Figure 6:**
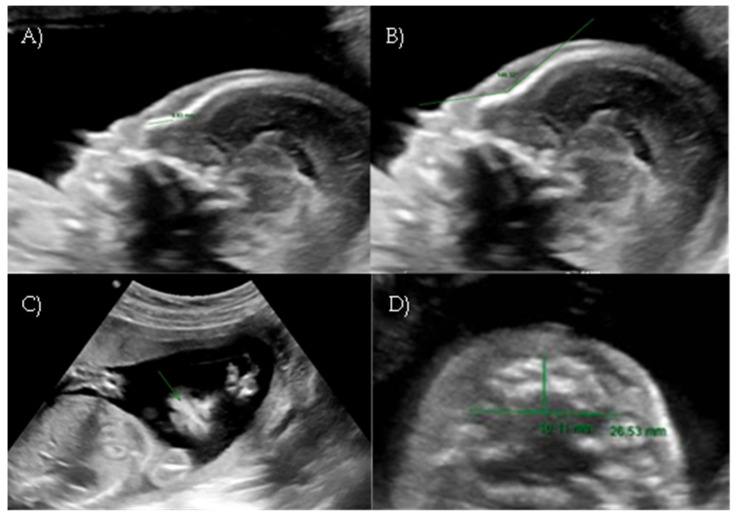
*Case 6.* (**A**) Flat fetal profile with verticalization of the nasal bone. (**B**) Nasal-frontal angle: 146.32°. (**C**) Absent nasolabial philtrum. (**D**) Flattening of the curvature of the maxillary alveolar processes. Width: 26.53 mm.

A targeted anomaly scan at our fetal medicine unit revealed a flat and dysmorphic fetal facial profile with verticalization of the nasal bone, increased NFA, and short columella, suggestive of BP. An associated feature was polyhydramnios. The ultrasound findings were suggestive of Binder-type maxillonasal dysplasia (Nasal-frontal angle: 146.32°, cut-off: <143°) such as the flattening of the curvature of the maxillary alveolar processes (Width: 26.53 mm, cut-off at 22 weeks: 23.9 mm) (Figure 6A–D).

Genetic investigations were recommended, including amniocentesis with standard karyotyping, QF-PCR, chromosomal microarray (CGH-array), and DNA storage. A fetal echocardiogram and genetic counseling were also advised. However, the patient declined invasive testing.

At 24 weeks of gestation intrauterine fetal demise (IUFD) occurred.

Fetal autopsy revealed a male fetus with craniofacial dysmorphism characterized by a short, markedly flattened nose, a short columella, and an acute nasolabial angle within the context of midface hypoplasia. There was also mild widening of the palpebral fissures with a normal intercanthal index (DICI). A protruding tongue was observed, giving the impression of macroglossia, though no cleft lip or palate defects were present. No other major macroscopic malformations were identified. The craniofacial abnormalities were consistent with a diagnosis of Binder-type maxillonasal dysplasia.

### 2.7. Case 7

A 34-year-old gravida 2 para 0 woman was referred at 20 weeks and 3 days of gestation following the detection of an abnormal fetal facial profile during a routine second-trimester ultrasound at our fetal medicine unit. The pregnancy was conceived spontaneously. A SARS-CoV-2 infection during the first trimester of pregnancy was reported.

First-trimester combined screening performed elsewhere was low risk for trisomy 21, 13, and 18.

**Figure 7:**
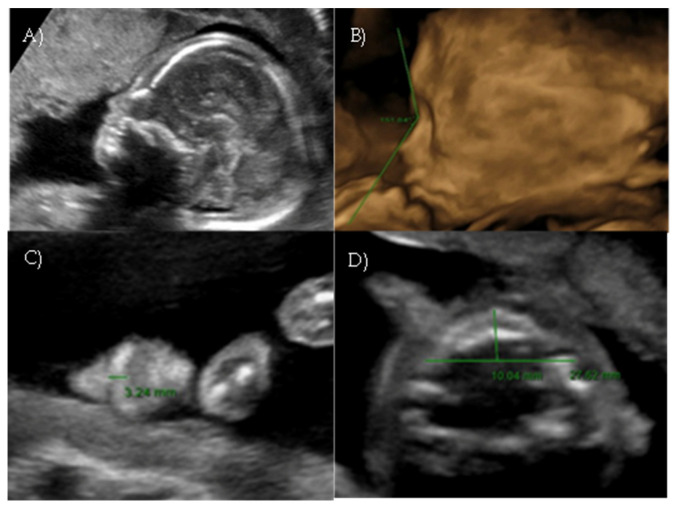
*Case 7.* (**A**) Flat fetal profile. (**B**) Nasal-frontal angle: 151.84°. (**C**) Absent nasolabial philtrum. (**D**) Maxillary width: 27.62 mm.

The routine anomaly scan was performed at our center at 20 weeks. A standard second-trimester ultrasound, following ISUOG guidelines 2022 [12], included visualization of the fetal facial profile in the midsagittal plane. The suspicion of an abnormal fetal profile was raised. Since a suspected craniofacial anomaly was present, a more detailed evaluation using axial, sagittal, and coronal planes was performed to assess the defect thoroughly by a fetal medicine expert. It revealed an isolated flat fetal facial profile suggestive of Binder-type maxillonasal dysplasia: the nasal-frontal angle measured 151.84° (cut-off <143°) and the maxillary width measured 27.62 mm (cut-off at 20 weeks: 22.4 mm) (Figure 7A–D).

Genetic investigations were recommended, including invasive testing with standard karyotype, QF-PCR, chromosomal microarray (CGH-array), and DNA storage. Standard and molecular fetal karyotypes were normal. The couple attended genetic counseling. Next-Generation Sequencing (NGS) and exome sequencing were discussed with the patients. However the couple declined further investigations due to the high cost.

The patient requested termination of pregnancy at 21 weeks.

Fetal autopsy confirmed an isolated severe hypoplasia of the maxillary bones, consistent with maxillonasal dysplasia (Binder type).

## 3. Discussion

Binder syndrome is a rare congenital facial anomaly characterized by an abnormal development of the nasal complex and the maxilla [6,20] occurring in less than 1 in 10,000 live births. The condition was initially described by Zuckerland in 1882 [21].

In 1962, Binder et al. [3] reported three cases and defined the phenotypic characteristics of the facial dysmorphism: a short nose with a flat nasal bridge, the absence or hypoplasia of the anterior nasal spine and columella, an acute naso-labial angle (76–88° vs. normal value 103–117°) with pronounced convexity of the upper lip, prognathism with tendency toward class III malocclusion due to maxillary hypoplasia, mild hypertelorism, and atrophy of the nasal mucosa. Depending on the severity of the syndrome, most patients have some or all of the characteristics [20,21,22,23,24,25].

Several publications indicate that ultrasound can detect the characteristic facial features of Binder phenotype before birth [6,7,8].

Our case series of seven fetuses diagnosed over a 22-month period at a single second-level center suggests that this condition may be more prevalent than previously thought in specialized prenatal diagnosis units.

However, rare congenital anomalies can appear more frequently within a specific time period due to a combination of factors, including environmental exposures, genetic factors, and chance occurrences [26,27,28,29,30].

Many congenital anomalies are considered sporadic, meaning they occur randomly without a clear cause. It is possible for these sporadic cases to cluster together by chance in a particular time or place, creating the appearance of a higher prevalence.

Furthermore, an apparent increase in the frequency of a rare fetal anomaly can indeed be a result of enhanced awareness and improved reporting by expert fetal medicine specialists, rather than a true rise in incidence [31,32,33].

Certain infections during pregnancy (Zika virus, cytomegalovirus) [34] can cause congenital anomalies. If an outbreak of such an infection occurs, it could lead to a cluster of related birth defects. We reported seven cases diagnosed during the COVID-19 pandemic of which one case occurred in a patient with COVID-19 infection during the first trimester. However, there is no evidence that exposure to COVID-19 infection or immunization during pregnancy increases the risk of congenital anomalies. The study from Magnus et al [35] is the largest, to date, of congenital anomalies after first-trimester infection with SARS-CoV-2 or COVID-19 immunization.

Using national birth registries, investigators identified 343,000 liveborn singleton infants conceived between March 2020 and February 2022. Rates of congenital anomalies among the 10,000 infants whose mothers were infected with SARS-CoV-2 in their first trimesters showed similar rates to those observed among infants whose mothers were not.

Among the 152,000 infants who were conceived after COVID-19 vaccines became available in January 2021, 29,000 infants whose mothers were vaccinated in the first trimester had a similar rate of congenital anomalies as those whose mothers were not vaccinated.

In our series three out of seven cases were linked to hyperemesis gravidarum. In the case of hyperemesis gravidarum, a decreased trend of admissions during the COVID-19 pandemic has been reported [36,37]. A study from Tieranu at al [38] reported that the admissions for hyperemesis gravidarum dropped dramatically, by 88% in 2020 and by 52% in 2021 and 2022. This has been attributed to the fear of going to the hospital, a common behavior in 2021–2022 due to possible hospital phobia, with delayed seeking of medical attention.

In 2000, Cook et al. [7] described for the first time the distinctive ultrasound signs of Binder syndrome found during routine second-trimester examination: midfacial hypoplasia with verticalized nasal bone, a short columella with flattened tip and alar wings, a nasofrontal angle (NFA) measuring >140 degrees, and maxillary retraction.

The next case was published in 2005 by Cuillier et al. [8].

In 2009, Levaillant et al. [6] proposed other ultrasound parameters suggestive of maxillonasal dysplasia. On the midsagittal plane, it is possible to calculate the nasofrontal angle (NFA), which is increased, with a value between 150 and 160°. On the transverse scan, a reduction in the convexity of the maxillary alveolar arch and flattening of its curve can be found, resulting in increased width, calculated according to Rotten’s formula [13]: MX = 0.75 × Gestational age + 7.41, with an average value of 23 mm at 21 weeks. Finally, on the coronal scan, there is disappearance of the labial filter and mild hypertelorism.

In 2010, Oztürk et al. [14] measured the nasofrontal angle (NFA) in 195 healthy fetuses between the 18th and 21st weeks of gestation, reporting minimum, maximum, and mean values of 110°, 143°, and 128°, respectively.

The median gestational age at diagnosis in our series was 21 weeks.

Our findings underline the importance of the identification of a normal fetal profile through an adequate ultrasound examination during the second trimester of pregnancy to exclude facial abnormalities that could signal various chromosomal or genetic syndromes.

According to ISUOG Guidelines [12] the basic examination of the face in the second trimester should include visualization of the upper lip, assessment of the presence and position of the orbits/eyes, and, if possible, assessment of the fetal profile.

If a facial anomaly is suspected, other anatomical landmarks, such as nose, nostrils, palate, maxilla, mandible, tongue, and ear position and size, need to be assessed.

The characteristic ultrasound features observed in our cases included flattened fetal facial profile, increased nasofrontal angle exceeding 143 degrees, verticalized nasal bones, and widened maxillary alveolar arch, consistent with established diagnostic criteria described by Levaillant et al. [13].

In our case series we report the first case described in the literature before 14 weeks during a routine first-trimester combined screening test for aneuploidies.

The 11 + 0- to 14 + 0-week scan provides an opportunity to assess fetal anatomy and should not be limited to assessment of fetal CRL and NT. Whilst cell-free (cf) DNA provides a highly effective means of screening for common aneuploidies, this test cannot identify structural defects.

Most structural anomalies occur in “low risk” pregnancies.

Evidence of normal anatomy at 11 + 0 to 14 + 0 weeks provides early reassurance for most pregnant women. Early identification of a major anomaly allows earlier genetic diagnosis and a wider window for parental counseling and decision making.

Several studies have shown that a systematic examination including a standardized protocol significantly increases the detection rate of anomalies in early gestation [39,40].

A systematic approach to detailed assessment of the fetal face at 11 + 0 to 14 + 0 weeks should include visualization of the fetal face in the midsagittal plane, which should be complemented with examination in either an axial or a coronal plane. The magnified midsagittal plane of the head and neck enables assessment of several anatomic regions of the face, including the forehead, nasal bone, maxilla, mandible, and mouth. Different facial angles and markers (e.g., maxillary gap, superimposed-line sign) have been proposed to assess the presence of facial clefts in the midsagittal view, but these need confirmation in other planes [41,42].

In an axial or coronal view an attempt should be made to visualize the eyes with their interorbital distance and the retronasal triangle, demonstrating the maxilla and the mandible. The nasal bone is “absent” or hypoplastic in 50–60% of fetuses with trisomy 21, and this can be used as an additional marker to improve efficacy of ultrasound-based screening.

A review of the literature on the first trimester period shows a knowledge gap on a comprehensive facial angle assessment for Binder syndrome diagnosis. While existing studies have extensively focused on nasal bone length measurements as primary screening markers for chromosomal abnormalities [43,44,45,46], particularly trisomy 21, we could not find a body of research specifically addressing the role of facial angles in detecting structural facial malformations such as Binder syndrome during early pregnancy.

The frontonasal angle, as established by Vicario et al. [47] in their study of 400 chromosomally normal fetuses, shows measurable parameters, with reproducible measurement techniques demonstrating high clinical reliability. However, these normative data have primarily been applied to chromosomal screening rather than structural malformation detection.

Zhou et al. [48] demonstrated that facial profile markers, including inferior facial angle and maxilla–nasion–mandible angle, shows a correlation with crown–rump length during the first trimester in Chinese populations, yet their clinical application remains limited to specific ethnic groups and focused primarily on chromosomal abnormalities rather than syndromic facial malformations.

The frontomaxillary facial angle, as validated by Plasencia et al. [49], shows high reproducibility in measurement with 95% of cases demonstrating differences within 3° between observers, suggesting that standardized facial angle assessment could be reliably implemented in clinical practice.

Present studies are not focused on measurements of facial angles specifically for early detection of Binder syndrome and other maxillonasal dysplasias. Our case series represents one of the few reports documenting prenatal diagnosis of Binder syndrome, yet comprehensive facial angle analysis remains underutilized in routine first-trimester screening protocols.

Future research should prioritize establishing standardized facial angle reference ranges specifically for detecting structural facial malformations, with particular emphasis on nasofrontal and frontomaxillary angles as potential screening markers for conditions like Binder syndrome in the first trimester.

We propose that integration of systematic facial angle assessment into first-trimester screening protocols [50] could significantly enhance early detection of facial malformations, enabling timely genetic counseling and appropriate pregnancy management. Advanced artificial intelligence algorithms and machine learning approaches, as suggested by emerging literature, may further optimize facial angle measurement accuracy and enhance detection of subtle facial dysmorphisms during routine first-trimester examinations.

The etiology of Binder Syndrome is not entirely clear. The etiology is heterogeneous, with multiple causative categories described: teratogenic/environmental, chromosomal (Xp22.3 deletion), and single gene disorders (autosomal recessive chondrodysplasia punctata, brachytelephalangic chondrodysplasia punctata, Conradi-Hunermann syndrome, Keutel syndrome, Stickler syndrome, Robinow syndrome, and infantile sialic acid storage disorder).

It is hypothesized to be a disorder of the prosencephalic center during embryonic growth. Conditions predisposing a patient to vitamin K deficiency, such as intractable vomiting, malabsorption disease, alcohol consumption during pregnancy, maternal treatment with warfarin or phenytoin, and autoimmune diseases such as LES or mixed connective tissue disease are considered possible etiological factors [29,30]. Several studies demonstrate that maxillonasal dysplasia is related to vitamin K deficiency, and the resulting abnormal formation of the vitamin K dependent matrix gamma/carboxyglutamic protein causes reduced growth of the embryonic nasal septum [1,31].

Over the years, a close association has been demonstrated between maxillonasal dysplasia and forms of punctate chondrodysplasia (CDP) [9], an heterogeneous group of congenital skeletal dysplasias characterized especially by periarticular and epiphyseal punctate calcifications, scoliosis, and long bone biometry below the 5th percentile. CDP may have a genetic cause with different patterns of inheritance [26,27].

In most cases, BP appears to occur sporadically. The genetic transmission of Binder syndrome has not been fully elucidated; however, several authors describe familial cases. For example, Olow Nordenram reported a family history in 36% of patients with BS, possibly explained by autosomal dominant, recessive, and X-linked transmission inheritance or association with multifactorial inheritance [28].

More than 50 conditions presenting with a flat profile have been described, although most of them have other associated anomalies [5].

Among genetic conditions that have low flat nasal bones apart from chondrodysplasia punctate are Keutel’s syndrome (hypoplasia of the distal phalanges, diffuse calcification of the ears, nose, and trachea), Apert’s syndrome (irregular craniosynostosis, short occipital–frontal diameter, flat occiput, ventriculomegaly, and syndactyly), Crouzon’s syndrome (craniosynostosis, brachycephaly, and short occipital–frontal diameter), Aarskog’s syndrome (brachycephaly and clinodactyly of the fifth finger), Rudiger’s syndrome (short digits), Robinow’s syndrome (short forearms, clinodactyly, and macrocephaly), Stickler’s syndrome (features of osteochondrodysplasia and congenital talipes), and chromosomal abnormalities, most commonly trisomy 21/18 [32,33].

Bosco et al., in 2024 [26], identified 47 cases of BP diagnosed antenatally from inception until 2024 in a literature review. A total of 12,8% of cases were isolated with no other associated anomalies at postnatal examination. A total of 19% of cases were possibly associated with non-hereditary conditions (hyperemesis gravidarum and maternal autoimmune diseases). Hereditary CDP was frequently associated (40%), with the X-linked CDP form 1 (CDPX1) being the most common. Authors recommended to investigate for CDPX1 (FISH for ARSE deletion) in male fetuses with isolated BP or with indicative findings and to offer exome sequencing where BP and other anomalies are found together.

These results highlight the importance of a detailed anatomical ultrasound evaluation when BP is diagnosed to ascertain or rule out associated anomalies underlying a syndromic condition.

A table (Table S1) summarizing similar cases of prenatally diagnosed Binder Phenotype reported in the literature in the past 15 years is available in the Supplementary Materials.

The etiology of BP in our series demonstrates the heterogeneous nature of this condition. Case 1 presented with a relevant familial history of craniofacial anomalies and hyperemesis gravidarum, suggesting possible genetic predisposition combined with maternal vitamin K deficiency secondary to intractable vomiting. Case 2 also experienced hyperemesis gravidarum in the first trimester, supporting the association between severe maternal vomiting and BP development as reported by Howe et al. [29] Case 5 involved a pregnancy complicated by mixed connective tissue disease and treatment with hydroxychloroquine, potentially linking autoimmune conditions to BP development. Case 7 reported SARS-CoV-2 infection during the first trimester. The remaining cases appeared to occur sporadically, which aligns with reports indicating that most BP cases arise without clear familial patterns as described by Keppler-Noreuil and Wenzel [1].

Our experience with invasive prenatal diagnosis revealed normal karyotypes and chromosomal microarrays in all three cases that underwent testing. This finding supports the observation that isolated BP is often associated with a normal chromosomal complement, distinguishing it from syndromic cases if linked with genetic abnormalities.

During the specified period, the local laboratory offered a comprehensive set of genetic tests, but Next-Generation Sequencing (NGS) and exome sequencing were not available locally and were thus not covered by the healthcare system if performed in a different region, placing the financial burden entirely on the patient. All patients undergoing amniocentesis declined further investigations.

The decision to decline invasive testing in four cases reflects the challenging balance between diagnostic certainty and parental preferences, emphasizing the critical role of comprehensive genetic counseling.

Parents need dedicated counseling with a multidisciplinary team to understand the diagnosis, potential implications, and treatment options, and be offered emotional support, enabling them to make informed decisions aligned with their values. It is crucial to rule out underlying genetic conditions and associated defects, which require genetic testing and close monitoring during pregnancy.

In fetuses with apparent isolated BP at midtrimester, genetic testing with chromosome microarray (CMA) and fluorescence in situ hybridization (FISH) for ARSE deletion can be offered for CDPX1 diagnosis while exome sequencing may be more informative in cases where there are other features suggestive of a genetic disorder or if family history indicates a possible inherited condition [26].

Fetuses with Binder syndrome are at increased risk for respiratory distress after birth due to a narrowed upper airway, which can lead to breathing and feeding problems.

Cervical vertebral anomalies have been found in approximately 50% of cases, especially defects of the anterior and posterior wall of the atlas, vertebral fusion, and persistence of the notochord [20,22]. Therefore individuals with Binder phenotype are at risk for several medical complications such as sleep apnea, mixed conductive and sensorineural hearing loss, and cervical spinal stenosis/instability.

To a lesser extent, it may be associated with other major malformations, particularly cardiac anomalies [2,7,8,23,24,25]. Intellectual disability does not seem to be a significant feature of Binder syndrome.

If it is an isolated finding, the prognosis for a fetus with Binder syndrome is generally good. Surgical treatments are available for Binder syndrome, often involving plastic surgery and orthodontic care to improve the facial structure and function.

However, the diagnosis of Binder syndrome is clinical, the differential diagnosis is large and although many of the diagnoses can be ruled out with further tests, others cannot. Hence the parents will be left with a degree of uncertainty that can be emotionally difficult to accept.

For pregnancies with Binder condition, standard obstetric care is advised, but with the crucial precautions of delivering at a tertiary medical center. Prenatal and perinatal care providers should be alerted to the associated conditions of Binder phenotype and mobilize appropriate resources to handle possible intubation in the immediate postnatal period. Postnatal issues, such as airway management and short- and long-term outcomes, are still difficult to predict with precision.

Pregnancy outcomes varied significantly in our cohort. Three patients opted for termination following diagnosis, one case resulted in intrauterine fetal demise, and one delivered prematurely with confirmed postnatal features. Two patients achieved term delivery: one case had a neonatal favorable outcome, the other case was complicated by neonatal respiratory distress at birth and feeding difficulties. Challenges in ensuring follow-up when patients attend different centers may further hamper detection rates.

## 4. Conclusions

The relatively high frequency of BP diagnosis at our center within a limited timeframe may reflect referral bias to a specialized center, but it also suggests that systematic evaluation of fetal facial anatomy during routine screening may identify more cases than previously found.

Our findings emphasize the importance of detailed ultrasound evaluation when BP is suspected, including assessment for associated anomalies that might suggest underlying syndromic conditions. However, the variability in pregnancy outcomes underscores the need for individualized counseling based on specific case characteristics and comprehensive genetic evaluation.

Early and accurate diagnosis enables appropriate genetic counseling, facilitates informed decision-making, and allows for multidisciplinary planning when pregnancy continuation is chosen.

Future research should focus on establishing standardized diagnostic criteria for prenatal BP diagnosis and conducting longitudinal studies to better define the spectrum of outcomes associated with this condition. The integration of artificial intelligence algorithms, particularly deep learning models like CNNs and U-Net, into clinical workflows shows promising potential for enhancing diagnostic precision and enabling more accurate early detection of fetal facial anomalies [51,52]. Additionally, investigation into the relationship between maternal conditions and BP development may provide insights into preventive strategies.

## Figures and Tables

**Table 1:** Summary of biometric data and characteristics of the seven cases.

Case	Referral GA	Nasofrontal Angle (NFA)	Nasal Bone Length (mm)	Maxillary Width (mm)	Additional Malformations	Genetic Findings	Pregnancy Outcome	Diagnosis
Case 1	21 weeks + 1 day	152.18°	7.0	-	None documented	Not investigated	VD at 40 weeks + 3 days	BP + Maternal chronic hypertension + Hyperemesis gravidarum
Case 2	13 weeks + 2 days	126.40°	- (4.2 17 + 4 w)	-	None documented	Normal karyotype, normal CGH-array	TOP at 19 weeks + 3 days	BP + Maternal hyperemesis gravidarum
Case 3	21 weeks + 2 days	151.50°	5.5	26.4	Left renal pelvis dilation (8 mm), Two echogenic intracardiac foci, Long bones at lower limits of normal	Normal karyotype, normal CGH-array	VD at 39 weeks + 6 days. Respiratory dis tress and feeding difficulties.	BP + Maternal hyperemesis gravidarum
Case 4	26 weeks	151.71°	- (8.22 32 + 1 w)	-	IUGR, Polyhydramnios, Long bones <5th percentile, Bilateral preauricular pits (postnatal)	Normal postnatal karyotype	Emergency cesarean at 35 weeks (PPROM)	BP + Maternal gestational diabetes
Case 5	21 weeks + 4 days	150.72°	6.9	27	None documented	Normal karyotype, normal CGH-array	TOP	BP + Maternal mixed connective tissue disease (hydroxychloroquine treatment)
Case 6	22 weeks	146.32°	5.8	26.5	Polyhydramnios	Not investigated	IUFD at 24 weeks	BP
Case 7	20 weeks + 3 days	151.84°	6.2	27.6	None documented	Normal karyotype, normal CGH-array	TOP	BP

## Data Availability

The data presented in this study are available on request from the corresponding author due to the privacy policies of the hospitals involved.

## Supplementary Materials

The following supporting information can be downloaded at: https://www.mdpi.com/article/10.3390/reports8030188/s1, Table S1: cases of prenatally diagnosed Binder Phenotype in the last 15 years.
