# Supplementary material for: Prenatal Ultrasound Diagnosis of Binder Phenotype: Case Series of Seven Patients and Literature Review
Source: Reports (MDPI). 2025 Sep 22;8(3):188. doi: 10.3390/reports8030188 (PMC12473893; doi:10.3390/reports8030188)
Supplement: Supplementary file 1 [file reports-08-00188-s001.zip › reports-3829770-supplementary.pdf]

Case Report

Prenatal Ultrasound Diagnosis of Binder Phenotype: Case Series of Seven Patients and Literature Review

Silvia Andrietti [1], Alessia Maccarrone [2], Giuseppe Gullo [3,*], Valentina Billone [3], Lina De Paola [4], Chiara Gaggero [1], Diliana Beleva [1], Chiara Calcagno [1] and Pierangela De Biasio [1]

1 Prenatal Diagnosis and Perinatal Medicine Unit, IRCCS Ospedale Policlinico San Martino, 16132 Genova, Italy; silvia.andrietti@gmail.com (S.A.); chiara.gaggero@hsanmartino.it (C.G.); diliana.beleva@hsanmartino.it (D.B.); chiara.calcagno2@hsanmartino.it (C.C.); pierangela.debiasio@hsanmartino.it (P.D.B.)

2 Department of Neurology, Rehabilitation, Ophtalmology, Genetics, Maternal and Infant Health (DiNOGMI), 16132 Genoa, Italy; alessia.maccarrone97@gmail.com

3 Department of Obstetrics and Gynaecology, Villa Sofia Cervello Hospital, University of Palermo,
90146 Palermo, Italy; valentina.billone@gmail.com

4 Department of Anatomical, Histological, Forensic and Orthopedic Sciences, Sapienza University of Rome, 00161 Rome, Italy; lina.depaola@uniroma1.it

* Correspondence: gullogiuseppe@libero.it

Table S1. cases of prenatally diagnosed Binder Phenotype in the last 15 years.

Case	Author, date	Maternal age (years)	Gestational age at diagnosis (weeks)	Genetic findings	Delivery mode	Prenatal associated findings	Postnatal/TOP associated findings	Neonatal complications	Possible causes
1	Alessandri, 2010	20	24	Normal	Term, vaginal	None	Short 3rd phalanx, SC		HG
2	Boulet, 2010		23	normal	Preterm, CS	SC femur, short 3rd phalanx	Multiple SC		CDPX1
3	Colin, 2012	25	18	Normal		Multiple SC	Multiple SC confirmation, short 3rd phalanx		maternal autoimmune disease (SLE) + epilepsy
4	Colin, 2012	26	14	Normal		Suspected CHD not otherwise characterized	none		maternal autoimmune disease (SLE) + tuberculosis
5	Toriello, 2013	22	17	Normal	Term, vaginal	Mild ventriculomegaly	Minor scoliosis at the age of 8	RDS	HG with vitamin K deficit-associated coagulopathy
6	Toriello, 2013	27	30	Normal	Term, vaginal	None	Short 3rd phalanx		HG
7	Toriello, 2013		20	Normal	Term, vaginal	Short long bones	Short long bones, SC epiphyses, periarticular SC		Maternal autoimmune disease (Crohn)
8	Ochiai, 2012	34	32	Normal	Preterm,CS	NT>99° centile at 1° trimester, low set ears, thoracic hypoplasia, finger contractures,cervicothoracic kyphosis, polyhydramnios, spinal canal stenosis	Short 3rd phalanx, multiple SC	RDS and others	CDPX1
9	Blumenfeld, 2016	29	22	Normal	Term, CS	None	bell-shaped thorax, multiple SC cervical spinal stenosis, small focus of gray matter heterotopia	RDS	CDPX2
10	Blumenfeld, 2016	34	26		Term, CS	SC femur	Telecanthus, bilateral epicanthal folds, overfolding of the right helix, multiple SC, coronal clefts in the lumbar vertebrae	RDS	CDPX2

11	Blumenfeld, 2016	27	21	Normal (done postnatally)	Preterm, vaginal	SC femur, ankle	Short fingers, multiple SC, cervical canal stenosis	RDS	unknown
12	Blask, 2018		21			None	SC hands and feet, short 3rd phalanx	Yes	Maternal autoimmune disease (SLE), CDPX1
13	Blask, 2018		23			None	SC femur, hands/feet, short 3rd phalanx	RDS	CDPX1
14	Blask, 2018		30			Small thorax	SC thumbs/feet, minimal short 3rd phalanx	RDS and others	CDPX1
15	Blask, 2018		19			None	Syndactyly right 4th and 5th toes	RDS and others	CDPX1
16	Blask, 2018		19			SC knee	Short 3rd phalanx	RDS and others	CDPX1
17	Blask, 2018		18			Multiple SC, Dandy-Walker malformation, mild hydrocephalus, VSD, persistent right umbilical vein, two lobed lungs	SC hands/feet, short 3rd phalanx		HG, CDXP1
18	Blask, 2018		26		TOP	None	Multiple SC		Unknown
19	Bosselut, 2019	34	21		Preterm, CS	Multiple SC, polyhydramnios	Multiple SC, short 3rd phalanx, strabismus (7yo)		Unknown
20	Bosselut, 2019	38	22		Preterm, Vaginal	SC tarsus, short 3rd phalanx	Short 3rd phalanx, SC femur, tarsus, strabismus (6yo)	RDS	CDPX1
21	Bosselut, 2019	37	22	Normal	TOP	SC femur, tarsus	Nd		Unknown
22	Bosselut, 2019	31	21	Normal	Term, vaginal	Choroid plexus cysts, SC coccyx, tarsus	Nd		Unknown
23	Bosselut, 2019	31	22	Normal	Term, vaginal	Bilateral talipes	Micrognatia, bilateral talipe, strabismus (8mo)		Unknown

24	Bosselut, 2019	33	28	Normal	Term, vaginal	None	Nd		Isolated BP
25	Bosselut, 2019	41	22	Normal	Term, vaginal	None	Learning difficulties (5yo)		Isolated BP
26	Bosselut, 2019	32	33	Normal	Term, vaginal	Multiple SC	Short stature		Unknown
27	Bosselut, 2019	45	24		Term, vaginal	SC tarsus, hyoid bone	Short 3rd phalanx, coronal lumbar and sacral vertebral cleft, multiple SC and sacral vertebrae, tarsus.	RDS	CDPX1
28	Bosselut, 2019	41	33	Normal	Term, vaginal	Multiple SC, short 3rd phalanx, persistent right umbilical vein, double right renal system	Short 3rd phalanx, cleft palate, SC		CDPX1
29	Bosselut, 2019	33	25	Normal	Term, vaginal	Short 3rd phalanx, multiple SC	Short 3rd phalanx, SC		CDPX1
30	Bosselut, 2019	31	23		Term, vaginal	Multiple SC	Short 3rd phalanx, multiple SC, upper thoracic vertebral cleft.		CDPX1
31	Bosselut, 2019	32	23		TOP	Multiple SC	Short 3rd phalanx, SC		CDPX1
32	Mazzone, 2019	24	23	Normal	TOP	SC, short limbs, short 3rd phalanx	SC including trachea, skeleton		CDPX1
33	Pop, 2020	39	21	Normal	Term, vaginal	None	None		Isolated BP
34	Veduta, 2021	34	25			None	None		Isolated BP
35	Mathonnet , 2021	34	18	Normal	TOP	Short 3rd phalanx, SC	Multiple SC including thyroid cartilage		Congenital vitamin K-dependent clotting factors deficiency (VKCFD gene)

36	Bosco, 2024		29	Normal	Preterm, CS	Polyhydramnios	Multiple SC, PFO, low set hears.	RDS and others	CDPX1
37	Sabu, 2024	28	26		Term, CS	Polyhydramnios	None		HG
38	Sabu, 2024	27	19		Term, vaginal	None	None		Physiognomy
39	Sabu, 2024	24	18		TOP	Hydrops, echogenic cardiac focus	Hydrops		Maternal autoimmune disease (overlap syndrome)
40	Sabu, 2024	25	19		Preterm	None	None		Maternal autoimmune disease (overlap syndrome)
41	Sabu, 2024	32	21		Term, CS	Mild hypertelorism, polyhydramnios, echogenic cardiac focus	None		Isolated BP
42	Sabu, 2024	26	21	Trisomy 21	TOP	Polyhydramnios, early IUGR, echogenic cardiac focus, echogenic bowel			Trisomy 21
43	Sabu, 2024	26	21	Normal kariotype and CMA	Ongoing pregnancy	echogenic cardiac focus			Probable physiognomy
44	Gatsis, 2025		22	Normal prenatal kariotype and CMA, postnatal WES: KMT2D mutation	Preterm, CS	SUA, ASD, ARSA, horseshoe kidney.	Blue sclera, Pulmonary hypertension, significant ASD, duplex right kidney.	Neonatal death	Kabuki syndrome

BP: Binder Phenotype; HG: hyperemesis gravidarum; TOP: termination of pregnancy; MRI: magnetic resonance imaging; CT: computerized tomography; RDS: respiratory distress syndrome. CDP: chondrodysplasia punctata; CS: C-section; VK: vitamin K; SC: stippled calcifications; SLE: Systemic Lupus Erythematosus; VSD: ventricular septal defects; PFO: patent foramen ovale, CMA: chromosomal micro-array; SUA: single umbilical artery; ASD: atrial septal defects; ARSA: aberrant right subclavian artery; WES: whole exome sequencing.
